# Identification of Hotspots in the European Union for the Introduction of Four Zoonotic Arboviroses by Live Animal Trade

**DOI:** 10.1371/journal.pone.0070000

**Published:** 2013-07-23

**Authors:** Benoit Durand, Sylvie Lecollinet, Cécile Beck, Beatriz Martínez-López, Thomas Balenghien, Véronique Chevalier

**Affiliations:** 1 Anses, Laboratoire de Santé Animale, Maisons-Alfort, France; 2 European Union Reference Laboratory for Equine Diseases, Laboratoire de Santé Animale, Maisons-Alfort, France; 3 VISAVET group, Animal Health Department, Veterinary School, Complutense University, Madrid, Spain; 4 UMR Contrôle des Maladies, Cirad, Montpellier, France; 5 AGIRs Unit, Cirad, Montpellier, France; Blood Systems Research Institute, United States of America

## Abstract

Live animal trade is considered a major mode of introduction of viruses from enzootic foci into disease-free areas. Due to societal and behavioural changes, some wild animal species may nowadays be considered as pet species. The species diversity of animals involved in international trade is thus increasing. This could benefit pathogens that have a broad host range such as arboviruses. The objective of this study was to analyze the risk posed by live animal imports for the introduction, in the European Union (EU), of four arboviruses that affect human and horses: Eastern and Western equine encephalomyelitis, Venezuelan equine encephalitis and Japanese encephalitis. Importation data for a five-years period (2005-2009, extracted from the EU TRACES database), environmental data (used as a proxy for the presence of vectors) and horses and human population density data (impacting the occurrence of clinical cases) were combined to derive spatially explicit risk indicators for virus introduction and for the potential consequences of such introductions. Results showed the existence of hotspots where the introduction risk was the highest in Belgium, in the Netherlands and in the north of Italy. This risk was higher for Eastern equine encephalomyelitis (EEE) than for the three other diseases. It was mainly attributed to exotic pet species such as rodents, reptiles or cage birds, imported in small-sized containments from a wide variety of geographic origins. The increasing species and origin diversity of these animals may have in the future a strong impact on the risk of introduction of arboviruses in the EU.

## Introduction

Emerging infectious diseases (EID) of human and animal have become a major concern in the past decades. The increasing occurrence of EID events [1] has been associated to the ongoing epidemiological transition (changes in patterns of diseases as societies develop) [2], a consequence of (i) the globalization of economic activities and cultures, (ii) the increasing rapidity and intensity of travel and distant contacts, (iii) the change in migration patterns [3], (iv) the intensification of urbanization, and (v) the climate change. EID events have been identified and characterized [1,4–6] and emergence mechanisms have been proposed and analyzed [7–12]. According to these studies, emerging pathogens are more often RNA viruses, zoonotic and/or vector-borne involving a broad host range. Since 2000, the European continent has faced a number of EID events caused by arboviruses, such as West Nile Virus (WNV) (lineage 1) in 2000 [13], Usutu virus in 2001 [14], WNV (lineage 2) in 2004 [15], bluetongue virus serotype 8 in 2006 [16] (as well as other BTV serotypes in the preceding years), Chikungunya in 2007 [17], Dengue in 2010 [18,19], and Schmallenberg virus in 2011 [20]. Some of these pathogen introductions have resulted in limited epidemics (Chikungunya, Dengue), other have given birth to large-scale epidemic waves (bluetongue serotype 8, Schmallenberg virus) [21]; some of these pathogens have become endemic in several parts of Europe (Usutu virus, WNV [22–24]). Pathogens are probably frequently introduced through the trade of live animals (or of products of animal origin) or through the arrival of infected arthropod vectors, most of these introductions being undetected [25]. In a recent prospective study conducted by the European Centre for Disease Prevention and Control, introduction of vector-borne diseases by global trade was one of the eight scenarios, considered plausible, of infectious disease threats facing the EU by 2020 [26].

Eastern and Western equine encephalomyelitis virus (EEEV and WEEV), Venezuelan equine encephalitis virus (VEEV) and Japanese encephalitis virus (JEV) are four zoonotic RNA arboviruses that may cause lethal encephalitis in human and horses. These diseases (Eastern and Western equine encephalomyelitis [EEE and WEE], Venezuelan equine encephalitis [VEE] and Japanese encephalitis [JE]) are considered emerging [1] and a recent prioritization study conducted in Europe ranked them among the 10 most important animal diseases and zoonoses [27]. Arboviruses introductions into previously free countries (or continents) thanks to international movements of persons are regularly reported (Chikungunya in Italy and Dengue in Croatia, France and Portugal (Madeira) [17–19]). Infected vectors transported with cargo may potentially allow the introduction of arboviruses in free areas (it is one of the possible modes of introduction of bluetongue virus serotype 8 in Northern Europe in 2006 [28–30]). Animal migrations may also support arbovirus introductions (bird migrations may explain WNV introductions into Europe [31]), as well as the trade of live animals (bird trade is one of the hypotheses that could explain the introduction of WNV in the Western hemisphere [32]).

Trade of live farm animals is a big business as, according to FAO, total export value represented US$ 16.5 billion in 2009 (FAOSTAT, http://faostat.fao.org consulted 2012-08-29). This total amount had doubled in ten years, with a total of US$ 8.7 billion in 1999. Total imports into European countries represented approximately half of this total: US$ 8.9 billion in 2009 (US$ 4.0 billion in 1999). Wildlife trade also represents an important market at the world level, with a total value estimated €406 million for live animal trade. The European Union (EU) is the world’s #1 importer for reptiles and cage birds [33].

Procedures for risk mitigation are routinely applied to secure the trade of live animals, both before the departure of the animals from their country of origin, and after their arrival in the EU: veterinary checks, establishment of health certificates, control of the origin of the imported animals, of their vaccination status (e.g. for equines and for VEEV), or quarantine in vector-free buildings (for cage birds, for example). However, these procedures may fail, and a residual risk of virus introduction persists by the trade of live animals. Live animals are not imported everywhere in the EU. The destination of farm animal consignments is linked to the spatial repartition of farms, and the destination of wild animal consignments to the geographic distribution of exotic pet buyers. For a given arbovirus, the presence of competent vectors and hosts in the local fauna also varies across the EU, as well as the density of susceptible hosts, that may reveal the presence of the virus. Therefore, for a given arboviral disease, the risk of introduction due to live animal imports and the potential consequences of such an introduction are likely to show strong geographic variations across the EU, depending on trade characteristics, local ecological conditions, and local population density.

This study was devoted to the residual risk (once risk mitigation procedures have been applied) posed by legal live animal imports into the EU (27 countries [EU27]) for the introduction of four arboviral diseases. The objectives were (i) to develop spatially explicit indicators of virus introduction risk and of the potential consequences of virus introductions, (ii) to map, using these indicators, the geographic variations of the introduction risk of EEEV, WEEV, VEEV and JEV, and (iii) to analyze the respective weights of farm animals and of exotic pets in this introduction risk.

## Materials and Methods

### Data

Live animal trade. Data were obtained from the European Commission (DG SANCO). The dataset was extracted from the TRACES (TRAde Control and Expert System) database, dedicated to the monitoring of live animals movements (and movements of animal products), between third countries and the EU (and within countries of the EU) (supporting information S1). The 2005-2009 subset of the database was filtered according to the country of origin and to the imported species group. For each virus, host species considered at risk were set according to literature: birds for WEEV; birds, rodents and reptiles for EEEV; equines, rodents and primates for VEEV; birds and swine for JEV (Table 1, Appendix S2). Before 2005, EEE, WEE, VEE and JE in horses belonged to the List B of the world organization for animal health (OIE) and the occurrence of equine clinical cases had thus to be reported by member states to the OIE. Since 2005, notifiable case definition has changed for JE and EEE, which are now OIE-listed “multiple species diseases”; whereas WEE and VEE remain OIE-listed “equine diseases”. Equines are dead-end hosts in the epidemiological cycle of the studied viruses (except for VEEV): horses may reveal virus circulation but are not necessary to this circulation. Furthermore, some level of underreporting certainly exists for OIE-listed diseases (e.g. avian influenza [34]:), especially for the former List B diseases, and underreporting level probably varies according to the country. For these reasons, rather than using OIE reports, consignment origins considered at risk were defined according to the 4 disease world repartition maps described in literature: the Americas for WEEV and EEEV, South America for VEEV, southern and eastern Asia for JEV (Table 1, Appendix S2). Origin and destination addresses of consignments were geocoded at the city level or more accurately, using GPS Vizualiser software, freely available (http://www.gpsvizualiser.com). In the few cases in which geocoding could not be achieved at the city level because of incomplete addresses, the corresponding origin or destination coordinates were set to the country or state capital. In TRACES, origin and destination addresses are given by the consignor, and it cannot be assumed that the points resulting from the geocoding process exactly correspond to the origin and destination of the imported animals. To take into account this probable imprecision, origin and destination points were discretized using a 10 km radius hexagonal grid covering the EU27. The coordinates of the origin and destination cells were computed for each consignment.

**Table 1:** Species group, geographic origin of the imported animals, and European vector species considered to analyze the introduction risk in the European Union of Eastern equine encephalomyelitis virus (EEEV), Western equine encephalomyelitis virus (WEEV), Venezuelan equine encephalitis virus (VEEV), and Japanese encephalitis virus (JEV).

Virus	Imported species group	Geographic origin	European vector species [39]
EEEV [49–51]	Rodents^4^, poultry^1^ and other birds^2^, reptiles^3^	Northern America, Central America, Caribbean, South America	*Culex* *pipiens* , *Aedes* *vexans* ^8^, *Aedes* *albopictus* ^9^
WEEV [52]	Poultry and other birds	Northern America, Central America, Caribbean, South America	*Aedes* *vexans* *, * *Aedes* *caspius* ^10^, *Aedes* *dorsalis* ^10^
VEEV [53]	Rodents^4^, Primates ^5^, Horses^6^	Central America, Caribbean, South America	*Aedes* *albopictus*
JEV [8]	Poultry^1^ and other birds^2^, Swine^7^	Southeastern Asia, Eastern Asia, India, Pakistan	*Culex* *pipiens* *, * *Aedes* *albopictus*

^1^ TRACES commodity code: 0105 (“Live poultry, that is to say, fowls of the species Gallus domesticus, ducks, geese, turkeys and guinea fowls”).

^2^ TRACES commodity code: 010631 (“birds of prey”), 010632 (“Psittaciformes, including parrots, parakeets, macaws and cockatoos”), and 010639 (“Other birds”).

^3^ TRACES commodity code: 010620 (“Reptiles, including snakes and turtles”).

^4^ TRACES commodity code: code 010619 (“Other mammals”) and “Rodentia” specified in taxonomic data.

^5^ TRACES commodity code: code 010611.

^6^ TRACES commodity code: code 0101 (“Live horses, asses, mules and hinnies”).

^7^ TRACES commodity code: code 0103 (“Live swine”).

^8^ Current taxonomic denomination: *Aedimorphus* *vexans* .

^9^ Current taxonomic denomination: *Stegomyia albopicta*.

^10^ Current taxonomic denomination: *Ochlerotatus* *caspius* and *Ochlerotatus* *dorsalis*

Land cover. Data were extracted from the CORIN (*Coordination de l’information sur l’environnement*) land cover (CLC) database, provided by the European Environment Agency [35]. The 44 classes of the CLC nomenclature aim at describing perennial structures of land occupation and are organized into a general-purpose 3-level hierarchy. We used the 2000 version of CLC, at a 1:250,000 working scale (resolution of 250 m). CLC data were discretized using the 10 km radius hexagonal grid covering the EU27. For each cell of this grid, we computed the proportion of the cell area covered by each of the 44 themes of CLC nomenclature.

**Figure 1:**
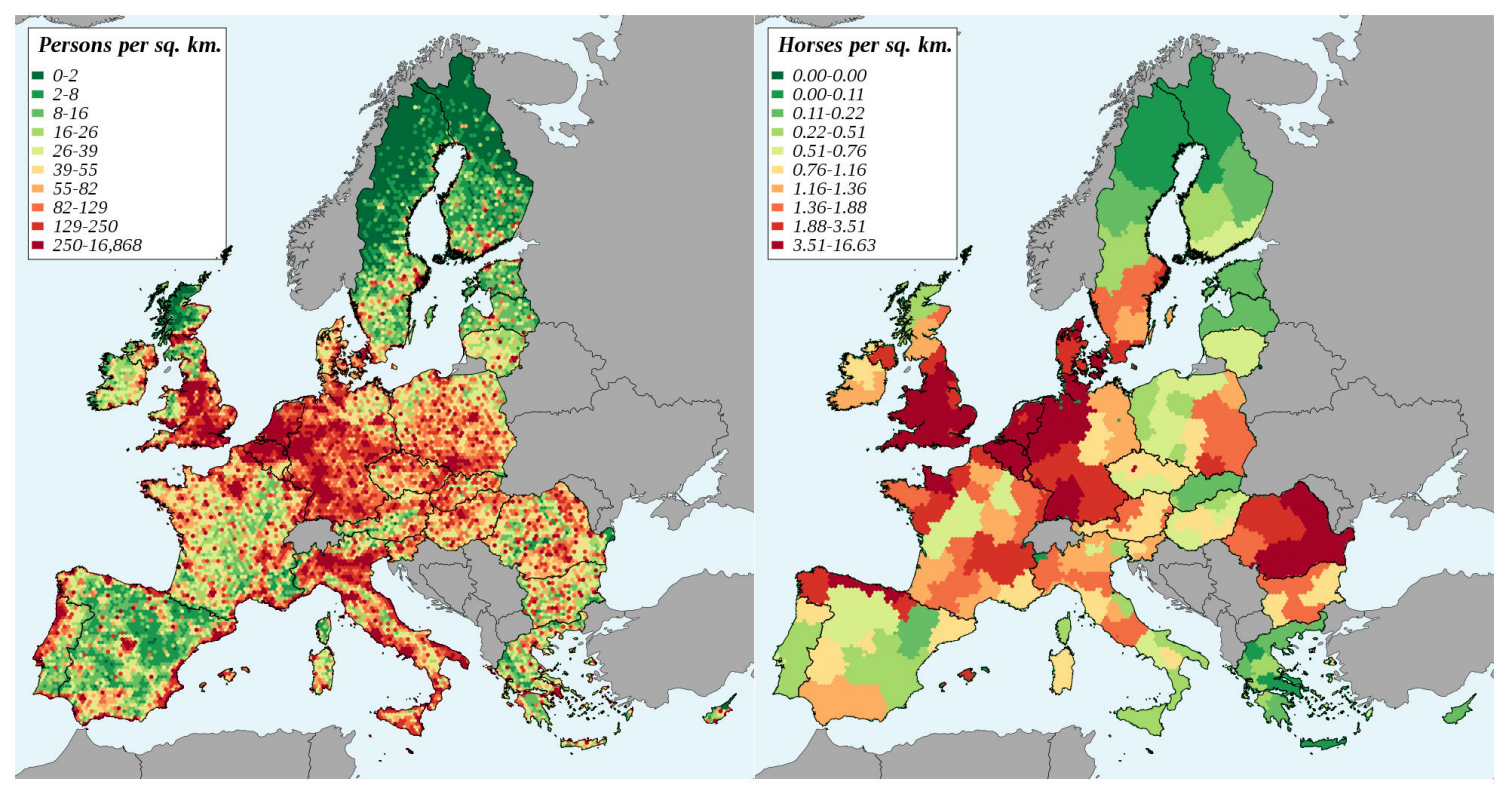
Population density of human (left) and of horses (right) in the European Union.

Population density. Population density data were extracted from the database “Population density grid of EU-27+” version 5 [36] provided by the European Environment Agency. This database is derived from the CLC database (2000 version) and contains the population density (number of inhabitants per square km) at a 1 km resolution for the EU27 and Croatia. This dataset was discretized using the 10 km radius hexagonal grid. For each cell of this grid, we computed the average population density (Figure 1, left). Horse population has not been quantified as precisely as human population, and several data sources were combined to derive realistic horse density data. The number of animals living in farms of each administrative area (NUTS 2 level) in 2007 was obtained from the EUROSTAT database (“Livestock: number of farms and heads by size of farm and NUTS 2 regions”, http://appsso.eurostat.ec.europa.eu/nui/show.do?dataset=ef_ls_ovaareg&lang=en, consulted 2012-08-27). However, this database only considers farm animals, and thus underestimates global horse population size. At the country level, global horse population estimates have been obtained in a recent study by Liljenstolpe [37]. These estimates were used to compute NUTS 2-level animal densities, assuming that the geographic distribution of horses housed in farms (EUROSTAT dataset) was representative of the global population. The resulting horse density map was discretized using the 10 km radius hexagonal grid. For each cell of this grid, we computed the average horse density (Figure 1, right).

### Indicator of virus introduction risk

The virus introduction risk depends on the number of imported animals and on their geographic origin. Areas considered at risk for consignment origins were the repartition areas of the four viruses described in literature (see Appendix S2). These areas are large and the studied viruses certainly do not circulate everywhere each year, but only seasonally, in specific but unknown sub-areas. Without an accurate knowledge of these areas, virus introduction risk is thus difficult to quantify precisely. However, it can reasonably be assumed that this risk increases with the diversity of the geographic origins of the consignments (within the geographic areas considered for virus introduction): when origins diversity increases, the probability that at least one consignment originates from an area where virus circulates also increases. If we assume that, in these areas, the infection prevalence has a comparable low level in reservoir species, the two major drivers of the introduction risk are the number of imported individuals and the diversity of their origins. Based on these assumptions, an empirical indicator *I*_*i*_, for virus introduction risk in a given 10 km diameter hexagonal cell was defined:

(1) $$I_{i}=\text{log}\left(N_{i}\right)\left(\text{1}+S_{i}\right)$$

where *N*_*i*_ is the total number of animals imported into cell *i* (log-transformed to buffer the large variations of consignment sizes according to the species), and *S*_*i*_ is the Simpson’s diversity index [38] of the consignment geographic origin for cell *i*:

(2) $$S_{i}=\text{1}-\frac{\sum n_{j}\left(n_{j}-\text{1}\right)}{N\left(N-\text{1}\right)}$$

where, for each possible origin cell *j*, *n*_*j*_ is the total number of animals imported from that cell. Simpson’s diversity index is the probability that two randomly taken imported animals originate from the same origin cell: it varies between 0 if all the imported animals come from the same cell, to 1 if each originates from a distinct cell.

The 2005-2009 cumulated value of the introduction risk indicator was computed for each cell of the 10 km diameter hexagonal grid covering the EU27, and for each of the four considered viruses.

### Potential consequences of virus introduction

Two potential consequences of virus introduction were successively analyzed: (i) the infection of local vectors by the imported animals, and (ii) the occurrence of clinical cases in human and horses. A scientific review was conducted by the European Food Safety Authority (EFSA) in 2009 to determine the potential European vector species for selected arboviruses [39]. According to this study, five European vector species were considered competent for one or several of the studied viruses: *Culex* *pipiens* , *Aedes* *vexans* , *Aedes* *albopictus* , *Aedes* *dorsalis* and *Aedes* *caspius* (Table 1). For each of the studied viruses and for a given 10 km hexagonal cell *i*, the potential infection of local vectors was quantified by the product of (i) the virus introduction risk, and (ii) the probability that a competent vector population is present in cell *i*:

(3) $$C_{i}=I_{i}\left[\text{1}-\prod_{v=\text{1}}^{V}\left(\text{1}-H_{v,i}\right)\right]$$

where *I*_*i*_ is the virus introduction risk for cell *i*, *V* is the total number of competent vector species, and *H*_*v,i*_ is the probability that at a given point inside cell *i*, a population of vector species *v* is present.

For a vector species and for a hexagonal cell, we used the proportion of the cell area covered by a suitable habitat as a proxy for the presence of a vector population. The calculation of this proportion was based upon land cover data and entomologist expert knowledge: for each vector species and for each CLC land cover theme, a three-level value was defined (non-suitable, moderately suitable, highly suitable) (Table 2). For a competent vector species *v*, the proportion *H*_*v,i*_ of the cell *i* covered by a suitable habitat was then:

**Table 2:** Suitability of land cover themes (CORINE nomenclature) for *Culex pipiens*, *Aedes caspius*, *Aedes dorsalis*, *Aedes vexans* and *Aedes albopictus* habitat (-: non-suitable: +/-: moderately suitable, +: highly suitable).

Land cover themes		*Cx* *. pipiens*	*Ae* *. caspius* *, Ae. dorsalis*	*Ae* *. vexans*	*Ae* *. albopictus*
Artificial surfaces	Urban fabric	+	-	-	+^c^
	Industrial, commercial, transport units	+	-	-	+^c^
	Mineral extraction sites	-	+/-	-	+^c^
	Dump sites	-	-	-	+^c^
	Construction sites	+	-	-	+^c^
	Artificial, non-agric. vegetated areas	+	-	-	+^c^
Arable land	Non-irrigated arable land	-	-	-	-
	Permanently irrigated land	+	-	-	-
	Rice fields	+	+/-	+	-
Permanent crops	Vineyards	-	-	-	-
	Fruit trees and berry plantations	+	-	-	-
	Olive groves	-	-	-	-
Pastures		-	-	+^a^	-
Heterogeneous agric.	Annual crops with permanent crops	-	-	-	-
areas	Other	+	-	-	-
Forests	Broad-leaved forest	-	-	+^a^	-
	Other	-	-	-	-
Scrub, herbaceous	Natural grasslands	-	-	+^a^	-
vegetation	Other	-	-	-	-
Open spaces with little or no vegetation		-	-	-	-
Inland wetlands	Inland marshes	-	+/-	+^b^	-
	Peat bogs	-	-	-	-
Maritime wetlands	Salt marshes, salines	-	+	-	-
	Intertidal flats	-	-	-	-
Inland waters	Water courses	+	-	+^b^	-
	Water bodies	+	-	+^b^	-
Marine waters	Estuaries	-	+	+	-
	Other	-	-	-	-

^a^ Only in the vicinity of inland marshes, water courses or water bodies

^b^ Only in the vicinity of pastures, broad-leaved forest or natural grasslands

^c^ Only inside administrative subdivisions where the presence of *Ae* *. albopictus* has been reported (ECDC-VBORNET)

(4) $$H_{v,i}=\frac{\text{1}}{Z}\sum_{k=\text{1}}^{M}m_{i,k}w_{v,k}$$

where *Z* is the number of pixels in a 10 km diameter cell, *M* is the number of land cover themes in CLC nomenclature, *m*_*i,k*_ is the number of cell *i* pixels that belong to land cover theme *k*, and *w*_*v,k*_=1 if the land cover theme *k* is a highly suitable habitat for the vector *v*, 0.5 if it is moderately suitable, and 0 if it is not suitable. Additional constraints (Table 2) were applied for *Ae* *. vexans* (neighbourhood between prairies or forests and water bodies or courses, because these landscapes are suitable for presence of *Ae* *. vexans* eggs only if they can be flooded) and for *Ae* *. albopictus* (administrative subdivisions where its presence has been reported, according to data collected by the European network of medical entomologists and public health experts VBORNET http://ecdc.europa.eu, consulted 2012-04-25).

The potential occurrence of clinical cases in human and horses was finally quantified by the product of (i) the potential infection of local vectors, and (ii) the local population density of disease-susceptible hosts. The 2005-2009 cumulated value of this indicator was computed for each 10 km hexagonal cell of a grid covering the EU27, for each of the four considered viruses.

### Numerical analysis

To identify regions with the highest introduction risk, virus-specific choropleth maps were drawn, based on the percentiles of the distributions of cell-specific indicator values. Maps were smoothed using an inverse distance weighting scheme. Global virus-specific relative risks were calculated by computing the sum (over the EU27) of the cell-specific indicator values, taking as a reference the value obtained for EEEV. The risk fraction attributable to each species or species group was computed by removing the corresponding consignments, and by computing a reduced risk using the resulting dataset. The risk fraction attributable to the species group was the difference between the total risk (computed using the complete dataset) and the reduced risk, divided by the total risk.

## Results

### Live animal imports

A total number of 150,154 vertebrate consignments entered the EU27 between 2005 and 2009, corresponding to 2.85 billion animals. Most of these were fishes: 2.78 billions animals against 65.7 millions of other vertebrates, corresponding respectively to 86,496 and 63,606 consignments. Among these consignments, 12,054 were considered for the risk of introduction of EEEV, WEEV, VEEV and JEV, corresponding to a total number of 22.5 million animals (Table 3). These were sent from 628 distinct hexagonal cells located in the Americas and Southeast Asia (Figures S1 and S2), and were delivered into 1,070 distinct cells in the EU27 (Figures S3 and S4). For WEEV, the vast majority of imports originated from countries that had reported disease cases to the OIE between 2005 and 2009 (100% for poultry and 91% for the other bird species). It was also the case for EEEV (100% for poultry, 91% for the other bird species, 95% for reptiles and 99% for rodents) and for JEV (84% for the other bird species, neither swine nor poultry imports occurred from countries considered for JEV introduction risk). This proportion was lower for VEEV as none of the imported equines originated from a country that had reported disease cases to the OIE between 2005 and 2009. However, this proportion was 30% for rodents and 81% for primates (World Animal Health Information Database, consulted 2012-08-27, http://www.oie.int/wahis_2/public/wahid.php/Wahidhome/Home).

**Table 3:** Total volume of live animal imports (heads, brackets: consignments) reported to the TRACES database between 2005 and 2009, for horses, swine, poultry, other birds, primates, reptiles and rodents.

Species	Origin of the animals							Total
	Europe	Africa	Asia		America		Australia	
			SE	Other parts	Northern	Southern	New Zealand	
Horses	75,043 (7,172)	2,118 (460)	43,035 (481)	4,698 (1,329)	15,496 (6,976)	15,703^3^ (2,025)	1,494 (681)	157,587 (19,124)
Swine	2,052 (31)	4 (2)	0^4^ (0)	0 (0)	2,332 (87)	0 (0)	0 (0)	4,388 (120)
Poultry	607,348 (101)	0 (0)	0^4^ (0)	214,054 (31)	15.5 M^1,2^ (518)	61,945^1, 2^ (7)	0 (0)	16.3 M^5^ (657)
Primates	934 (62)	17,460 (225)	23,682 (209)	613 (26)	518 (22)	667^3^ (21)	2 (2)	43,876 (567)
Other birds	384,025 (424)	622,995 (444)	16,309^4^ (79)	14,020 (109)	391,348^1, 2^ (169)	49,209^1, 2^ (160)	2,062 (12)	1,479,968 (1,397)
Reptiles	74,935 (234)	1,570,393 (1,897)	820,150 (623)	84,702 (127)	5,881,321^1^ (2,580)	427,529^1^ (605)	42 (1)	8,859,072 (6,067)
Rodents	132,795 (822)	17,743 (100)	239,506 (642)	6,915 (109)	215,780^1^ (5,879)	448^1, 3^ (11)	608 (63)	613,795 (7,626)
Total	1,277,132 (8,826)	2,230,713 (3,128)	1,142,682 (2,034)	325,002 (1,731)	22 M (16,231)	549,871 (2,829)	4,208 (759)	27.5 M (35,558)

^1^ Consignments considered for EEEV emergence risk

^2^ Consignments considered for WEEV emergence risk

^3^ Consignments considered for VEEV emergence risk

^4^ Consignments considered for JEV emergence risk

^5^ Millions

### Indicator of virus introduction risk

**Figure 2:**
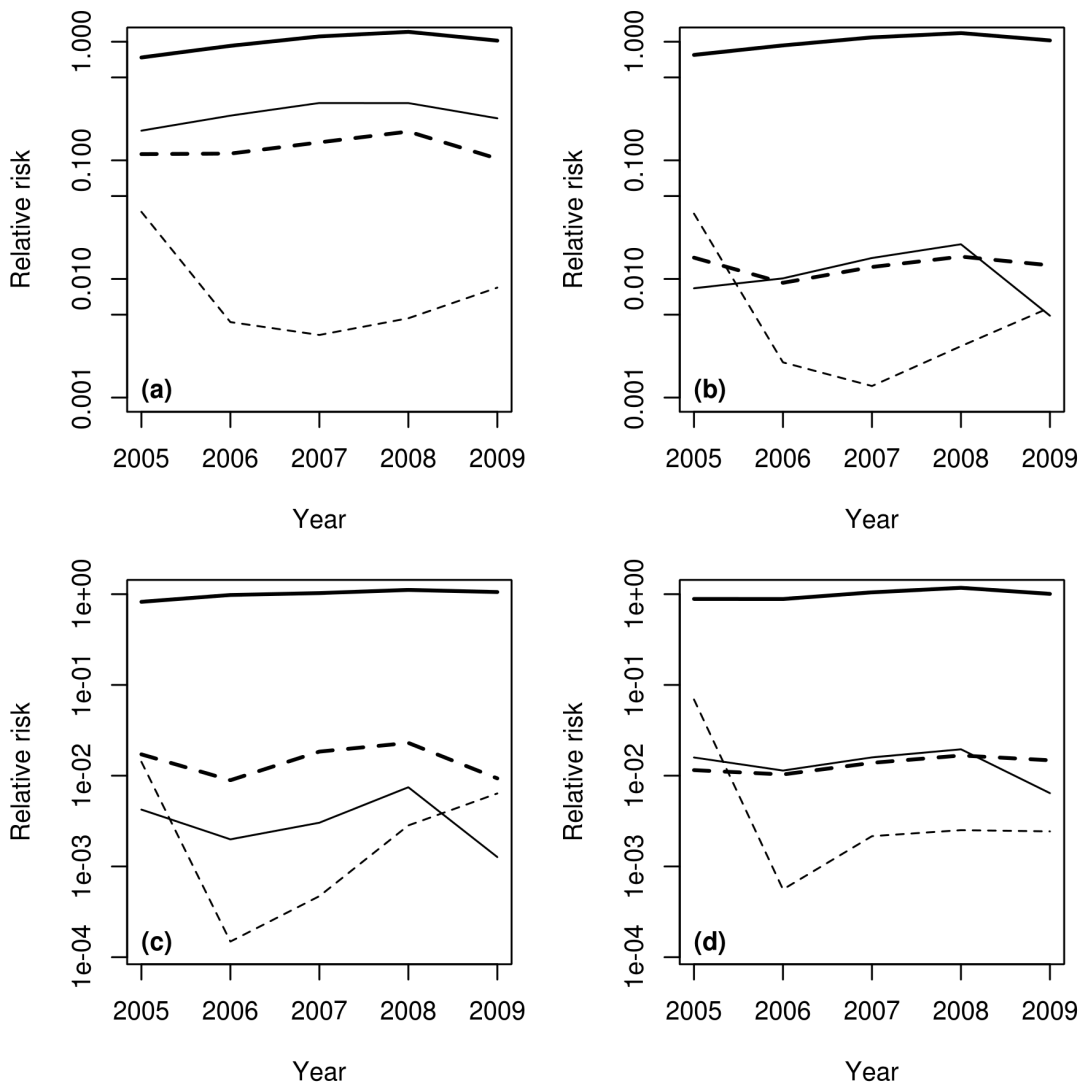
Evolution of virus introduction risk and of the potential consequences of virus introductions in the European Union, 2005-2009. (a) relative risk of virus introduction (reference: average value for EEEV), (b) potential infection of local vectors (reference: average value for EEEV), (c) potential occurrence of clinical cases in human (reference: average value for EEE), (d) potential occurrence of clinical cases in horses (reference: average value for EEE). Thick plain lines: Eastern equine encephalomyelitis virus, thin plain lines: Western equine encephalomyelitis virus, thick dashed lines: Venezuelan Equine encephalomyelitis, thin dashed lines: Japanese encephalitis. Y-axes are logarithmic.

**Figure 3:**
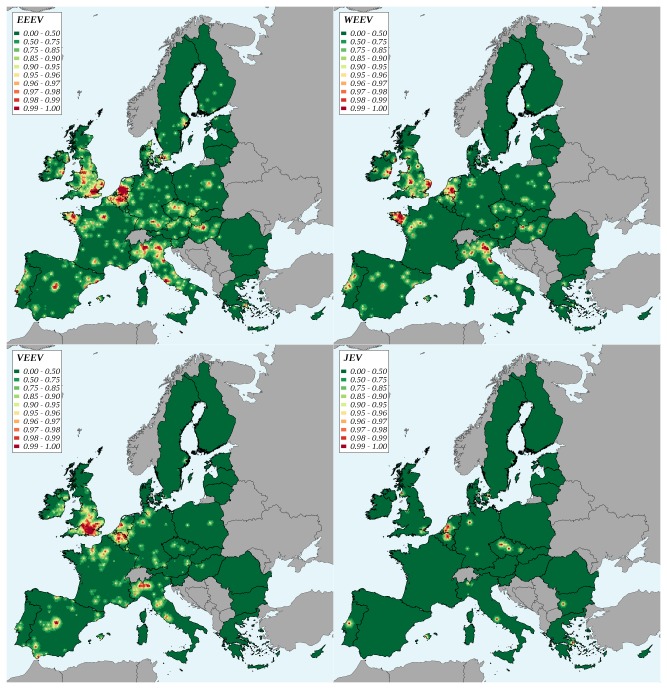
Geographic variations of the virus introduction risk in the European Union, 2005-2009. EEEV: Eastern equine encephalomyelitis virus, WEEV: Western equine encephalomyelitis virus, VEEV: Venezuelan equine encephalomyelitis virus, JEV: Japanese encephalitis virus. Classes are percentiles of the distribution.

At the level of the EU27, the introduction risk was highest for EEEV. Taking EEEV as a reference, this introduction risk was approximately 3 times lower for WEEV, 5 times lower for VEEV and 50 times lower for JEV (Table 4). Introduction risk appeared relatively stable over time for EEEV, WEEV and VEEV. A marked decline was observed for JEV between 2005 and 2006, and the risk level increased slowly afterwards (Figure 2). For EEEV, approximately 70% of the introduction risk was attributable to exotic pets (rodents, birds other than poultry, reptiles). The opposite result was obtained for WEEV and VEEV with, respectively, 71% and 98% of introduction risk attributable to non-pet species (poultry for WEEV, mainly horses for VEEV) (Table 4). As neither poultry nor swine were imported from Southeast Asia, 100% of JEV introduction risk was attributable to birds other than poultry. Marked geographic variations of introduction risk indicator were observed (Figure 3). The risk map appeared patchy, areas with the highest risks (99th percentile of the distribution) were located in the Netherlands (EEEV, WEEV, JEV), Belgium (EEEV, VEEV, JEV), the south of England (VEEV, EEEV), the north of Italy (EEEV, WEEV, VEEV), and the west of France (EEEV, WEEV). All over the EU, country capitals and major cities were associated to higher values of introduction risk indicator.

**Table 4:** Global virus introduction risk, potential infection of local vectors, and fraction attributable to the imported species for Eastern equine encephalomyelitis virus (EEEV), Western equine encephalomyelitis virus (WEEV), Venezuelan equine encephalomyelitis virus (VEEV), and Japanese encephalitis virus (JEV), European Union, 2005-2009.

	Species	Virus introduction risk and potential infection of local vectors							
		EEEV		WEEV		VEEV		JEV	
		Intro. ^a^	Vectors^b^	Intro.	Vectors	Intro.	Vectors	Intro.	Vectors
Global risk		1.00	1.00	0.37	0.02	0.17	0.02	0.02	0.02
Attributable risk^c^	Horses					95%	95%		
	Swine							0%	0%
	Poultry	24%	17%	71%	66%				
	Primates					3%	1%		
	Other birds	6%	5%	26%	26%			100%	100%
	Reptiles	35%	35%						
	Rodents	22%	25%			2%	4%		
	Non-pets^d^	24%	17%	71%	66%	98%	96%	0%	0%
	Exotic pets^e^	72%	78%	26%	26%	2%	4%	100%	100%
	All	100%	100%	100%	100%	100%	100%	100%	100%

^a^ Relative risk of virus introduction (reference: EEEV)

^b^ Potential infection of local vectors (reference: EEEV)

^c^ Risk difference computed with and without the considered species group. As for two distinct species, shipments destination areas may overlap, the column sums (for the eight species groups or for pets and non-pets) may not be 100%.

^d^ Horse, swine, poultry and primate

^e^ Birds other than poultry, reptiles, rodents

### Potential consequences of virus introduction

**Figure 4:**
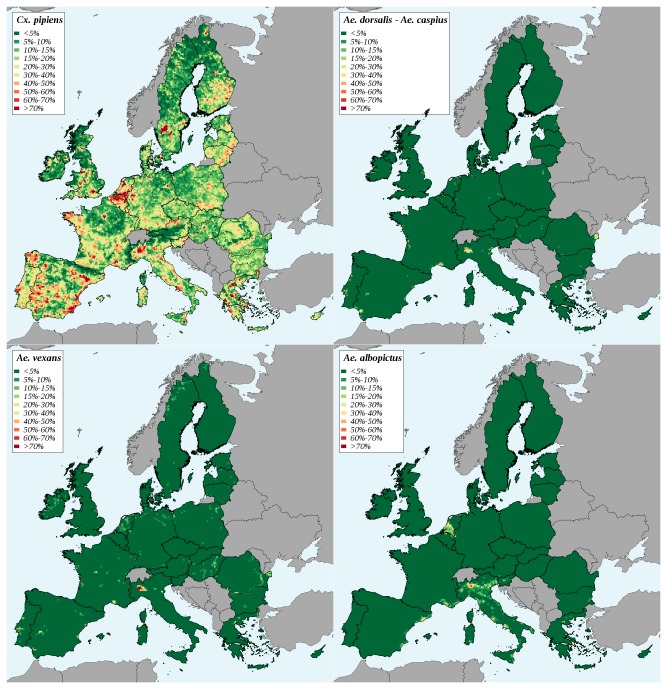
Proportion of land surface covered by a suitable habitat for competent vector species in the European Union. *Culex* *pipiens* : Eastern equine encephalomyelitis virus and Japanese encephalitis virus. *Aedes* *dorsalis* , *Aedes* *caspius* : Western equine encephalomyelitis virus. *Aedes* *vexans* : Eastern and Western equine encephalomyelitis viruses. *Aedes* *albopictus* : Eastern equine encephalomyelitis virus, Venezuelan equine encephalomyelitis virus and Japanese encephalitis virus (only administrative areas of the EU where it the presence of *Ae* *. albopictus* has been reported are considered).

**Figure 5:**
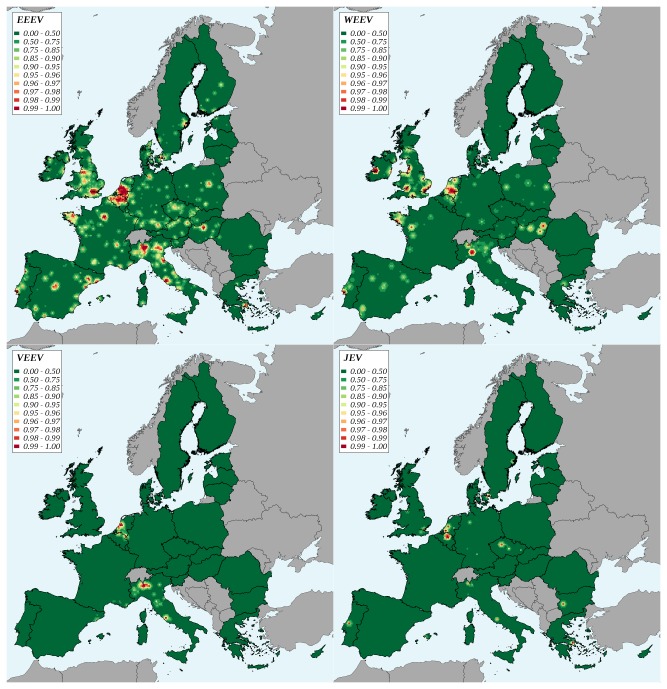
Geographic variations of the potential infection of local vectors after a virus introduction in the European Union, 2005-2009. EEEV: Eastern equine encephalomyelitis virus, WEEV: Western equine encephalomyelitis virus, VEEV: Venezuelan equine encephalomyelitis virus, JEV: Japanese encephalitis virus. Classes are percentiles of the distribution.

*Culex* *pipiens* is the most common mosquito species in Europe. As expected, most of the EU land surface contained suitable habitats for this mosquito species (Figure 4). For *Ae* *. caspius* and *Ae* *. dorsalis* , favourable areas corresponded to the major European wetlands. It was also the case for *Ae* *. vexans* , for which part of the valleys of the main rivers (e.g. Rhine, Danube) were also favourable. For *Ae* *. albopictus* , suitable habitats corresponded to urbanized areas in Italy, the Netherlands and along the Mediterranean coast. The differences between the studied arboviruses were more marked for the potential infection of local vectors than for the introduction risk: the total value obtained for WEEV, VEEV and JEV were much lower than that of EEEV (Table 4). Variations of risk by year were similar to those of the introduction risk (Figure 2), the VEEV and WEEV curves being closer than for the introduction risk. For EEEV, approximately 80% of potential infection of local vectors was attributable to exotic pet imports. Oppositely, for WEEV and VEEV, the fraction attributable to non-pet species imports was dominant with 66% and 96% of total risk, respectively (Table 4). For EEEV, the main area with a high potential for the infection of local vectors was the north of Belgium and the south of the Netherlands (Figure 5). The north of Italy (Po valley, Milan and Venice areas) also presented a high potential for the infection of local vectors. The same areas showed high risks for the infection of local vectors by WEEV, VEEV and JEV. Large cities were often affected, especially for EEEV and WEEV.

**Figure 6:**
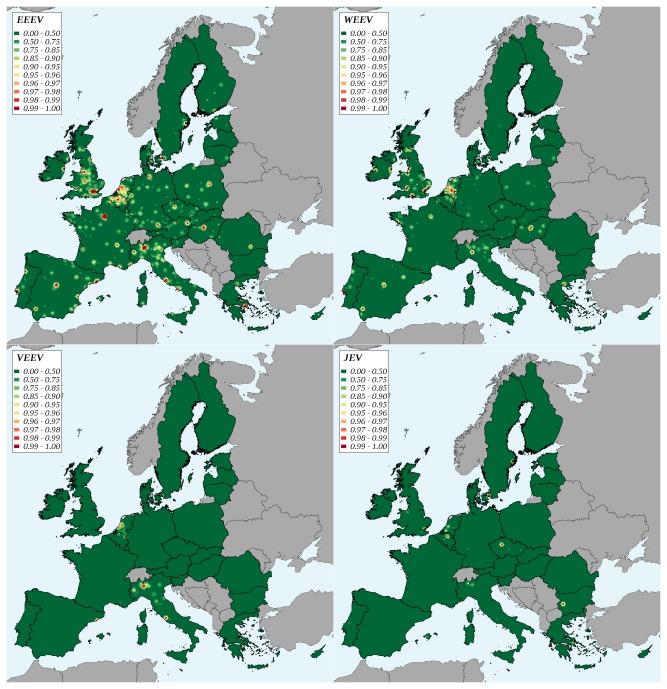
Geographic variations of the potential occurrence of clinical cases in human after a virus introduction in the European Union, 2005-2009. EEE: Eastern equine encephalomyelitis, WEE: Western equine encephalomyelitis, VEE: Venezuelan equine encephalomyelitis, JE: Japanese encephalitis. Classes are percentiles of the distribution.

**Figure 7:**
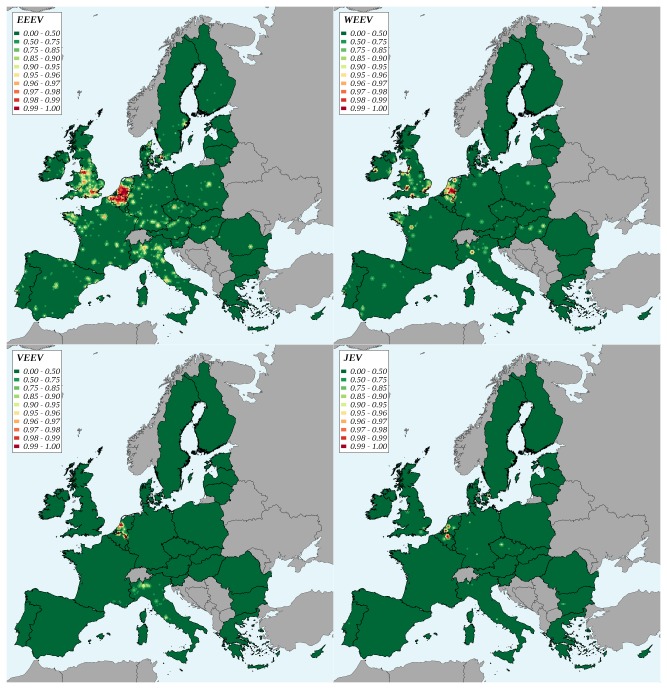
Geographic variations of the potential occurrence of clinical cases in horses after a virus introduction in the European Union, 2005-2009. EEE: Eastern equine encephalomyelitis, WEE: Western equine encephalomyelitis, VEE: Venezuelan equine encephalomyelitis, JE: Japanese encephalitis. Classes are percentiles of the distribution.

The overall potential for the occurrence of clinical cases in human and horses was much lower for WEE, VEE and JE than for EEE. The respective roles of the imported species appeared contrasted. For EEE, the fraction attributable to exotic pets was dominant (91% and 81% for the potential occurrence of clinical cases in human and horses, respectively). Oppositely, for VEE, the fraction attributable to non-pet species was dominant (99%). The situation was intermediate for WEE: the fraction attributable to exotic pet species was slightly higher for the risk of potential occurrence of clinical cases in human (53%), whereas in horses, most of the risk was attributable to non-pet species (62%) (Table 5). For human, the potential occurrence of clinical cases remained approximately constant for EEE, VEE and WEE between 2005 and 2009; the level being always higher for VEE than for WEE. For JE the indicator value strongly decreased from 2005 to 2006 but increased afterwards, until reaching in 2009 a level close to that of 2005 (Figure 2). Similar trends were observed for the potential occurrence of clinical cases in horses, VEE and WEE presenting however similar levels, and JE a stable level since 2007. High potential areas for the occurrence of clinical cases in human (Figure 6) coincided with major cities, the Benelux countries and the north of Italy presenting a higher risk for each of the four viruses. For the occurrence of clinical cases in horses (Figure 7), Belgium and the Netherlands were clearly the highest risk areas, for each of the four viruses.

**Table 5:** Global potential occurrence of clinical cases in human and horses, and fraction attributable to the imported species for Eastern equine encephalomyelitis (EEE), Western equine encephalomyelitis (WEE), Venezuelan equine encephalomyelitis (VEE), and Japanese encephalitis (JE), European Union, 2005-2009.

	Species	Potential occurrence of clinical cases (reference: EEE)							
		EEE		WEE		VEE		JE	
		Human	Horses	Human	Horses	Human	Horses	Human	Horses
Global risk		1.00	1.00	0.01	0.02	0.03	0.02	0.01	0.03
Attributable risk^a^	Horses					99%	98%		
	Swine							0%	0%
	Poultry	4%	15%	43%	62%				
	Primates					<1%	<1%		
	Other birds	3%	6%	53%	35%			100%	100%
	Reptiles	36%	39%						
	Rodents	27%	20%			2%	1%		
	Non-pets^b^	4%	15%	43%	62%	99%	99%	0%	0%
	Exotic pets^e^	91%	81%	53%	35%	2%	1%	100%	100%
	All	100%	100%	100%	100%	100%	100%	100%	100%

^a^ Risk difference computed with and without the considered species group. As for two distinct species, shipments destination areas may overlap, the column sums (for the eight species groups or for pets and non-pets) may not be 100%.

^b^ Horse, swine, poultry and primate

^e^ Birds other than poultry, reptiles, rodents

## Discussion

Geographic risk variations showed the existence of hotspots in the EU for the introduction of EEEV, WEEV, VEEV and JEV by live animal trade. The existence of such hotspots highlights the need for an increased awareness and for an adaptation of surveillance strategies. Parts of Belgium, of the Netherlands and of the north of Italy were among the highest risk areas in the EU27 for each of the four studied viruses. These areas are densely urbanized, and received many consignments of exotic pet species. Belgium and the Netherlands are also major poultry breeding areas that received numerous consignments of poultry species. Northern Italy was the sole area of the EU that appeared suitable for each of the five considered vector species. Belgium and the Netherlands appeared particularly suitable for *Cx* *. pipiens* , and also, at a lesser extent, for the two considered *Aedes* species. Finally, these areas are among the most densely populated of the EU, especially Belgium and the Netherlands that also have a dense horse population. The combination of these factors explains the higher introduction risk as well as the more severe potential consequences of a virus introduction. It is worth noting that, besides the four considered viruses, these hotspots are consistent with recent emergences of arboviruses in the EU: bluetongue serotype 8 in Belgium and in the Netherlands [40], Schmallenberg virus in Germany and in the Netherlands [20], Usutu and West-Nile viruses in northern Italy [17,22,41], where recent laboratory results also suggest a possible circulation of JEV [42].

Three main drivers may explain the observed geographic variations of the introduction risk and of the potential consequences of virus introductions: (i) the import of host species that may introduce the virus (which determines the virus introduction risk), (ii) the potential European vector species and the spatial variations of habitat suitability for these species (which determines the potential infection of local vectors), and (iii) the population density of human and equine (which determines the potential occurrence of clinical cases). Results show that none of these three drivers was sufficient to explain, alone, the geographic variations of the risks, which rather resulted from their combination, with varying results according to the area in the EU. Of the four studied pathogens, the highest introduction risk and the most severe potential consequences were obtained for EEEV. This predominance is first explained by the host species considered for EEEV introduction: rodents, birds and reptiles. Most of the EEEV introduction risk, and most of the potential consequences of such introductions, was attributed to exotic pet species. In 2005-2009, the most numerous animals imported into the EU belonged to poultry species, which arrived in the EU in large size consignments, from a limited number of geographic origins. Besides, exotic pet species such as rodents, cage birds or reptiles, that could also support EEEV introduction, were delivered in EU countries in smaller size consignments, sent from much more varied origins. Considering that the virus introduction risk depends not only on the number of imported animals but also on the diversity of consignments origins, species imported in numerous small size shipments from many different origins may induce similar risks as those imported in large consignments from a limited set of areas. Furthermore, host species was linked to the destination of consignments. Poultry consignments were delivered in major European production areas, which are mainly non-urban, whereas exotic pets were mainly delivered in large cities, where they are commercialized. EEEV introduction risk thus applied both in urban and in non urban areas. The species considered for potential infection of local vectors also explained the predominance of EEEV. Among these species, *Cx* *. pipiens* had a very large repartition that could allow local populations to be infected in most of the places where EEEV could be introduced, especially in urban areas. The high population density in such areas finally explains the higher risk for the occurrence of EEE clinical cases.

For WEEV, VEEV and JEV, the introduction risk and the potential consequences of virus introductions were lower than for EEEV. The introduction risk of WEEV by poultry or exotic bird consignments was widespread, but each of the species considered for the potential infection of local vectors had a narrower repartition than *Cx* *. pipiens* . This constrained the potential infection of vectors by WEEV. For VEEV, the introduction risk was almost exclusively attributed to horses, and was also widely spread. However, none of the imported horses originated from a country that had reported clinical cases between 2005 and 2009: VEEV introduction risk was thus probably overestimated. Furthermore, *Ae* *. albopictus* , considered for the potential infection of local vectors, was limited to Italy and to the Netherlands. In the Netherlands, *Ae* *. albopictus* has been regularly observed in glasshouses, although surveillance data suggest that the species has not established itself in the surrounding areas [43]. Thus, the potential infection of local vectors by VEEV was probably overestimated in this area. Conversely, for JEV, the limiting factor was not the vector species ( *Culex* *pipiens* ), but rather the volume of imports of birds from Southeast Asia (no swine was imported from this area), that strongly decreased after 2005 because of influenza bans.

Our results are coherent with a recent qualitative analysis of the emergence risk of zoonotic arboviruses by trade and migration [44] according to which the chances for establishing new endemic foci were moderate for VEEV and for JEV, and moderate to high for WEEV and EEEV. To our knowledge, no quantitative analysis has been conducted on the introduction risk in the EU for EEEV, WEEV, VEEV or of JEV. This can be explained by the lack of quantitative data about the prevalence of these viruses in their maintenance hosts in their natural repartition areas that makes the parameterization of quantitative risk assessment models difficult. The approach proposed here does not aim at estimating introduction probabilities, but risk indicators that vary in the same way as the actual risk and may be used to compare the risk levels for different pathogens or for different geographic areas. The whole methodology is generic and could be easily applied to other arboviruses. The proposed indicator for risk introduction combines the number of imported animals and the diversity of their geographic origins. Assuming that each origin has a low probability of harbouring an enzootic virus circulation, the introduction risk is increased by the diversity of origins. This diversity was quantified using Simpson’s index, which is widely used in biodiversity studies. In our case, this index takes into account not only the diversity of consignments origins, but also the repartition of the imported animals in these consignments. For a given number of origins and of imported animals, Simpson’s index value will be the greatest if each origin provided the same number of animals. Indeed, this corresponds to the worst case situation, in terms of virus introduction risk. The potential infection of local vectors by the introduced animals was quantified using an original model of habitat suitability for potential European vector species that combines land cover data available all over the EU (Corin Land Cover) with entomologists’ knowledge about the suitability of land cover themes for each of the considered vector species. A similar work has been recently performed to identify European suitable areas of Rift valley fever circulation given a viral introduction [45]. Four of the five mosquito species considered here were studied, namely *Cx* *. pipiens* , *Ae* *. vexans* , *Ae* *. albopictus* and *Ae* *. caspius* . The species distributions, determined according to entomologists’ knowledge, were validated using trapping data from Italy. A good consistency was observed between the expected distribution and field data. We may thus assume that our maps of expected presence of vectors are relatively accurate and close to field reality. In the future, besides land cover, the proposed distribution maps could be improved by integrating other environmental factors such as latitude, elevation or climatic variables such as temperature or rainfalls. Seasonal variations of vector abundance in the EU may influence the potential infection of local vectors by introduced animals, according to the calendar date at which they are imported. It is also the case for the seasonal variations of vector abundance in the country of origin that may influence the risk of infection of imported animals, and thus the introduction risk. Moreover, the local availability of competent hosts (birds and rodents) and host-feeding patterns of mosquito have to be considered. For example, *Cx. Pipiens* is known to be mainly a bird-feeder, *Ae* *. caspius* and *Ae* *. dorsalis* are rather mammal-feeders and *Ae* *. albopictus* is rather opportunistic. Thus, the potential infection of local vectors may be changed depending on the interaction between imported host species and vector species. For simplicity reasons, host-feeding patterns were not considered here, and this could have induced an overestimation of the potential infection of local vectors.

Only the risk of virus introduction by legal live animal trade was considered in the present study. Risk mitigation measures were not taken into account and the calculated risk indices thus correspond to a residual risk, once the mitigation procedures have been performed. However, at least in principle, these procedures are equally applied all over the EU, and thus do not bias the geographic variations of risk. Illegal trade is, by definition, difficult to quantify [46]. However, even if the individual market value of the illegally imported animals may be very high, the total number of imported animals is probably much lower than the total number of legally imported animals. Besides the legal or illegal trade of live animals, other introduction pathways are possible for the studied pathogens: the movements of persons and of cargo. International transportation authorities estimated that 831 million passengers flew internationally in 2007, and the world tourism organization estimated 924 million tourists’ arrivals in 2008. The European Union (EU) was the first destination of these tourists: among the 10 most visited countries in 2006, 6 belonged to the EU, which represented 30% of the total number of tourists’ arrivals [47]. Human is considered a dead-end host for EEEV, WEEV and JEV. However, viraemic persons could represent an introduction path for VEEV. The movement of approximately 90% of non-bulk cargo worldwide is carried out by containers on specific transport ships. The movements of goods, with the generalization of this containerization, have been responsible for the transcontinental movements of vector species, such as *Ae* *. albopictus* , from south-eastern Asia to northern America, southern America and Europe [30]. Introduction of infected vectors in cargo containers is thus also possible. However, the respective importance of live animal trade and of persons and cargo movements for the introduction of EEEV, WEEV, VEEV and JEV still needs to be determined.

Data about the international trade of exotic pets are scarce, and this work is the first quantitative description of exotic pet imports in the EU. An unexpected large number of exotic pets imports was observed: reptiles and rodents as well as, at a lesser extent, cage birds. Rodents were the second most imported species group in terms of number of consignments (after horses), while reptiles were the second most imported species group in terms of number of animals (after poultry). The major difference between exotic pet trade and farm animal trade is of course the diversity of species. Four species dominate farm animals trade (cattle, pig, poultry and horses), whereas the trade of live wild animals (sold as exotic pets) involves hundreds of species (ornemental fishes, reptiles, cage birds and birds of prey, mammals), a diversity that is favoured by the use of internet by buyers. This species diversity is unfortunately poorly documented as the identity of the imported species is not (or rarely) indicated in the TRACES database. Officials that control the consignments at the border inspection posts should be better trained for reptile and cage bird species identification [46]. Moreover, if regulations of international farm animal trade have been designed to protect the disease-free status of importing countries, it is not the case for the regulations of the international trade of wild animals, which have rather been designed to protect endangered species [48]. At their arrival in the EU, housing conditions and health status of such imported animals shoud thus be more systematically controlled. Finally, arboviruses are known to often have a broad host range, a characteristic which may be explained by a positive selective pressure [7] especially in a context of global biodiversity loss. This diversity is not fully documented for existing arboviruses and field studies regularly allow identifying new host species in natural foci (e.g., for EEEV [49–51]). In the future, the increasing species diversity of imported animals may thus have an important impact on the introduction of arboviruses.

## Supporting Information

Appendix S1 Live animal imports into the European Union. (DOC)

Appendix S2 Eastern and Western equine encephalomyelitis, Venezuelan equine encephalitis and Japanese encephalitis. (DOC)

Figure S1 Origin of horses, swine, poultry and primate imported into the European Union, 2005-2009. (TIF)

Figure S2 Origin of birds other than poultry, reptiles and rodents imported into the European Union, 2005-2009. (TIF)

Figure S3 Destination of live animal imports that could have allowed EEEV and WEEV introduction in the European Union, 2005-2009. EEEV: rodents, poultry, other birds and reptiles imported from the Americas. WEEV: poultry and other birds imported from the Americas.
(TIF)

Figure S4 Destination of live animal imports that could have allowed VEEV and JEV introduction in the European Union, 2005-2009. VEEV: horses, rodents, and primates imported from South America (including Central America and the Caribbean). JEV: birds other than poultry imported from Southeast Asia (including Japan, Korea, China, India and Pakistan).
(TIF)
