# Supplementary material for: Identification of Hotspots in the European Union for the Introduction of Four Zoonotic Arboviroses by Live Animal Trade
Source: PLoS One. 2013 Jul 23;8(7):e70000. doi: 10.1371/journal.pone.0070000 (PMC3720944; doi:10.1371/journal.pone.0070000)
Supplement: Appendix S1 — (DOC) [file pone.0070000.s001.doc]

**Identification of hotspots in the European Union for the emergence of four zoonotic arboviroses after viral introduction by live animal trade.**

Benoit Durand^1,*^, Sylvie Lecollinet^1,2^, Cécile Beck^1,2^, Beatriz Martínez-López^3^, Thomas Balenghien^4^, Véronique Chevalier^5^

^1^Anses, Laboratoire de Santé Animale, Maisons-Alfort, France

^2^European Union Reference Laboratory for Equine Diseases, Laboratoire de Santé Animale, Maisons-Alfort, France

^3^VISAVET group, Animal Health Department, Veterinary School, Complutense University, Madrid, Spain

^4^UMR Contrôle des Maladies, Cirad, Montpellier, France

^5^AGIRs Unit, Cirad, Montpellier, France

^*^Corresponding author, e-mail: benoit.durand@anses.fr

# Appendix S1. Live animal imports into the European Union

## TRAde Control and Expert System

TRACES (TRAde Control and Expert System) is an integrated system dedicated to the monitoring of live animals movements (and movements of animal products), between third countries and the EU (and within countries of the EU). Its construction was decided by the Commission Decision 2003/623/EC, and TRACES started functioning in 2004. Registered consignors use the system to fill online import certificates that are validated by local official veterinary services. These certificates are then checked at the EU border inspection posts, where officials who inspect the animals use TRACES for issuing a Common Veterinary Entry Document (CVED), sent to the veterinary authority of the Member State of destination. The CVED follows the consignments in the EU (if its entry is not rejected) and can then be consulted during checks carried out en route and/or at the destination. CVED includes a number of information including the entry date, the border inspection post, the consignor country and address, the destination country and address, the number of animals, a commodity code, and, optionally, taxonomic information: type, class, family and/or species.

## Imports of primates, horses, swine, rodents, birds or reptiles between 2005 and 2009

A total of 35,558 entries to the EU27 were recorded between 2005 and 2009, consignments corresponding to a total of 27.5 million animals. A satisfactory geocoding precision was obtained: 96% of origin addresses and 99.6% of destination addresses could be geocoded at the city level. Poultry represented 59% of the 27.5 million imported vertebrates other than fishes, followed by reptiles (32%) and by birds other than poultry (5%) (Table 3). However, in terms of consignments, poultry and swine were the less frequently imported animals (<1000 consignments in 5 years), the most frequently imported animals being horses (54% of consignments), rodents (21% of consignments) and reptiles (17% of consignments). Poultry and swine had the narrowest geographic origin: swine came exclusively from European and North American countries (four wharthogs [*Phacochoerus africanus*] were imported from Tanzania); it was also the case for most of poultry shipments, some originating however from Russia and from South America (Table 3, Fig. S1). Consignments of other species or species groups (horses, primates, birds other than poultry, reptiles and rodents) originated from the five continents, although the total number of shipments originating from Australia and New Zealand was quite limited (Table 3, Fig. S1 and S2). The main origins were Europe and southeast Asia for horses; southeast Asia and Africa for primates; Africa, North America and Europe for birds other than poultry; North America and Africa for reptiles; and southeast Asia and North America for rodents (Table 3, Fig. S1 and S2).

Within the EU, the destination of poultry shipments from the Americas reflected the location of the main breeding areas and concerned most of the EU countries, with strong variations in the amount of imported animals. It was also the case for the birds other than poultry imported from the Americas. Destination of reptile imports from this area appeared again widely spread, but with lower differences in the number of imported animals (Fig. S3). Destination of horses arriving from South America reflected the location of breeding areas, training centres and hippodromes. Rodents and primates originating from South America had only a limited number of destinations. It was also the case for birds other than poultry imported from Southeast Asia, with a concentration of these destinations in Benelux countries (Belgium, The Netherlands and Luxembourg) (Fig. S4).
