# Supplementary material for: Identification of Hotspots in the European Union for the Introduction of Four Zoonotic Arboviroses by Live Animal Trade
Source: PLoS One. 2013 Jul 23;8(7):e70000. doi: 10.1371/journal.pone.0070000 (PMC3720944; doi:10.1371/journal.pone.0070000)
Supplement: Appendix S2 — (DOC) [file pone.0070000.s002.doc]

**Identification of hotspots in the European Union for the emergence of four zoonotic arboviroses after viral introduction by live animal trade.**

Benoit Durand^1,*^, Sylvie Lecollinet^1,2^, Cécile Beck^1,2^, Beatriz Martínez-López^3^, Thomas Balenghien^4^, Véronique Chevalier^5^

^1^Anses, Laboratoire de Santé Animale, Maisons-Alfort, France

^2^European Union Reference Laboratory for Equine Diseases, Laboratoire de Santé Animale, Maisons-Alfort, France

^3^VISAVET group, Animal Health Department, Veterinary School, Complutense University, Madrid, Spain

^4^UMR Contrôle des Maladies, Cirad, Montpellier, France

^5^AGIRs Unit, Cirad, Montpellier, France

^*^Corresponding author, e-mail: benoit.durand@anses.fr

# Appendix S2. Eastern and Western equine encephalomyelitis, Venezuelan equine encephalitis and Japanese encephalitis

## Repartition areas

EEEV, WEEV and VEEV are alphaviruses (*Togaviridae* family). EEEV infections in equines have been recognized in Cuba for more than 50 years. EEEV circulates in western hemisphere, from Argentina to Canada, and in North America, mainly in the eastern side. In North America, EEEV is probably the most virulent alphavirus [1] with very high case-fatality rates in human (50-75%) and in horse (70-90%). Significant mortality can also be observed in swine and in domesticated birds (e.g. pheasants, emus). In Central and South America, EEEV is mainly an equine pathogen [2]. WEEV is a recombinant virus that combines EEEV-like and Sinbis virus-like genomic sequences [3]. It circulates in most of the American continent, from the western parts of Canada to Argentina. Case-fatality rates are lower than for EEEV both in human (3-7%) and in horse (3-50%) [1]. Disease incidence has decreased since the 1950's, this decline being rather due to factors affecting human and horse exposure than to an attenuation of strain virulence [4]. In the Americas, VEEV is the most important human and equine alphavirus, in terms of morbidity and of mortality. Some epidemics have caused hundreds of thousands of equine and human cases. In endemic areas, VEEV also causes a significant number of clinical cases, part of human cases being probably confounded with Dengue [2]. VEE viruses form a complex including epizootic strains (serotypes IAB and IC) and enzootic strains (several species separated into six subtypes) [5]. The fact that enzootic and epizootic strains only differ by a single amino-acid substitution [6] explains the recurrence of VEE epidemics in Central and Southern America. JEV is the major arbovirus causing encephalitis syndromes in the world. It belongs to the *Flaviviridae* family (genus *Flavivirus*), and has a wide geographic distribution as it circulates in eastern and southern Asian countries, from Japan to India and Pakistan [6]. The disease is endemic in these countries and constitutes an important public health issue with approximately 60,000 cases per year, and a 25% fatality rate in children [7]. Approximately 50% of patients develop neurological after-effects. An effective vaccine exists for humans and is largely used, in particular in Vietnam.

## Vertebrate hosts

Reservoir and amplification hosts of EEEV are passerines, and recent data indicate that some rodent species also play this role [8]. Snakes may also develop significant and prolonged viraemia, and could be overwintering hosts [9,10]. Humans, horses and swine are considered dead-end hosts. It is also the case for WEEV, of which maintenance and amplification hosts are birds in which infection is unapparent [1]. Several species or rodents are reservoir hosts in VEEV enzootic cycle. Enzootic VEEV strains generate little or no viraemia in experimentally infected horses, and are thought unable of causing epidemics. They are present in sylvatic cycles in several tropical and subtropical regions of South and Central America as well as in Mexico and Florida. On the contrary, humans and horses develop a high titre viraemia following the infection with epizootic strains, and thus serve as efficient amplifying hosts. Despite the dramatic consequences of infection, humans are considered dead-end hosts for JEV, and its maintenance hosts are pigs and water birds. Chickens and ducks are also implied in the cycle. Pigs develop a high and prolonged viremia. They are the principal amplifying hosts and may act as a bridge between birds and humans [6].

## Vectors

*Culex pipiens* is an ubiquitous mosquito. It presents a great ecological plasticity allowing it to colonize both rural and urban areas. *Aedes dorsalis* and *Aedes caspius* are very close species that have similar habitats (estuaries, marshes, water bodies, rice fields), *Ae*. *dorsalis* being rather found in northern Europe, and *Ae. caspius* in southern Europe. *Aedes vexans* lays its eggs in moist mud of draining ponds; it is thus mostly found in rice fields and in flooded areas (pastures, grasslands or forest areas), where it can be locally very abundant. *Aedes albopictus* is adapted to man-made breeding sites and in Europe, it is mainly an urban mosquito, the resistance of its eggs to cold and desiccation and their ability to be transported in tyres or 'lucky bamboo' plants, allowing it to colonize areas. In Europe, *Ae. albopictus* first emerged in Albania in 1979, and progressively spread in neighbouring countries (Greece, Montenegro, Bosnia Herzegovina, Croatia, Slovenia, Italy, France, Spain) [11] and in the Netherlands, in 2005 [12]. As for several other vectors, data about its geographic expansion are centralized by the European network for arthropod vector surveillance for human public health (VBORNET consortium). Repartition maps are regularly prepared that aggregate presence data using EU administrative subdivisions (NUTS 3 level).

# References

[1] Zacks MA, Paessler S (2010) Encephalitic alphaviruses. Vet Microbiol 140: 281-286.

[2] Weaver SC, Winegar R, Manger ID, Forrester NL (2012) Alphaviruses: population genetics and determinants of emergence. Antiviral Res 94: 242-257.

[3] Hahn CS, Lustig S, Strauss EG, Strauss JH (1988) Western equine encephalitis virus is a recombinant virus. Proc Natl Acad Sci U S A 85: 5997-6001.

[4] Forrester NL, Kenney JL, Deardorff E, Wang E, Weaver SC (2008) Western Equine Encephalitis submergence: lack of evidence for a decline in virus virulence. Virology 380: 170-172.

[5] Weaver SC, Ferro C, Barrera R, Boshell J, Navarro J-C (2004) Venezuelan equine encephalitis. Annu Rev Entomol 49: 141-174.

[6] Weaver SC, Barrett ADT (2004) Transmission cycles, host range, evolution and emergence of arboviral disease. Nat Rev Microbiol 2: 789-801.

[7] Erlanger TE, Weiss S, Keiser J, Utzinger J, Wiedenmayer K (2009) Past, present, and future of Japanese encephalitis. Emerg Infect Dis 15: 1-7.

[8] Arrigo NC, Adams AP, Watts DM, Newman PC, Weaver SC (2010) Cotton rats and house sparrows as hosts for North and South American strains of eastern equine encephalitis virus. Emerg Infect Dis 16: 1373-1380.

[9] White G, Ottendorfer C, Graham S, Unnasch TR (2011) Competency of reptiles and amphibians for eastern equine encephalitis virus. Am J Trop Med Hyg 85: 421-425.

[10] Graham SP, Hassan HK, Chapman T, White G, Guyer C, Unnasch TR (2012) Serosurveillance of eastern equine encephalitis virus in amphibians and reptiles from Alabama, USA. Am J Trop Med Hyg 86: 540-544.

[11] Medlock JM, Hansford KM, Schaffner F, Versteirt V, Hendrickx G, Zeller H, Bortel WV (2012) A review of the invasive mosquitoes in Europe: ecology, public health risks, and control options. Vector Borne Zoonotic Dis 12: 435-447.

[12] Scholte E-J, Jacobs F, Linton Y-M, Dijkstra E, Fransen J, Takken W (2007) First Record of Aedes (Stegomyia) albopictus in the Netherlands. European Mosquito Bulletin 22: 5-9.
