# Supplementary figures and images for: Identification of Hotspots in the European Union for the Introduction of Four Zoonotic Arboviroses by Live Animal Trade
Source: PLoS One. 2013 Jul 23;8(7):e70000. doi: 10.1371/journal.pone.0070000 (PMC3720944; doi:10.1371/journal.pone.0070000)

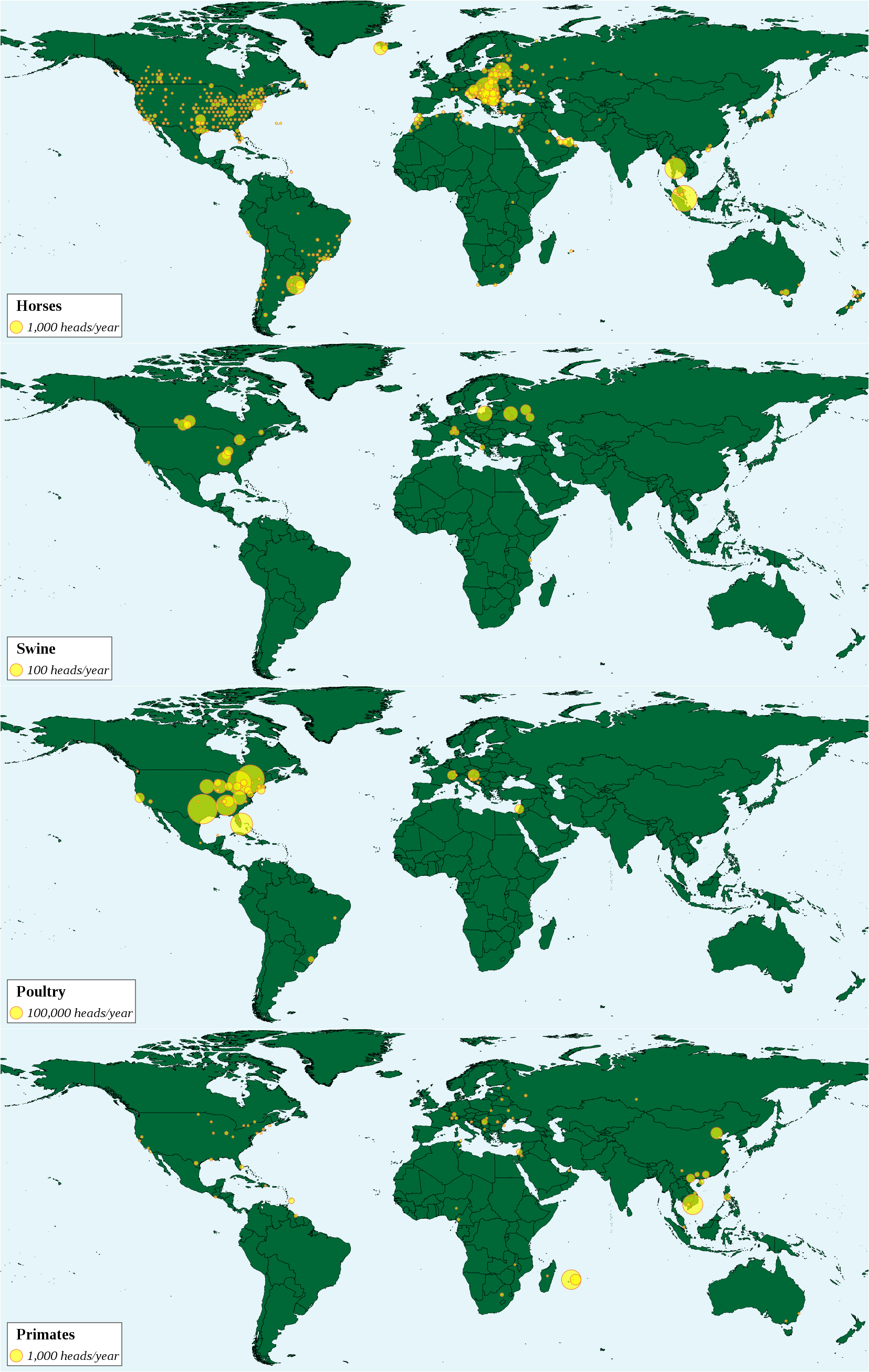

Supplement: Figure S1 — (TIF) [file pone.0070000.s003.tif]

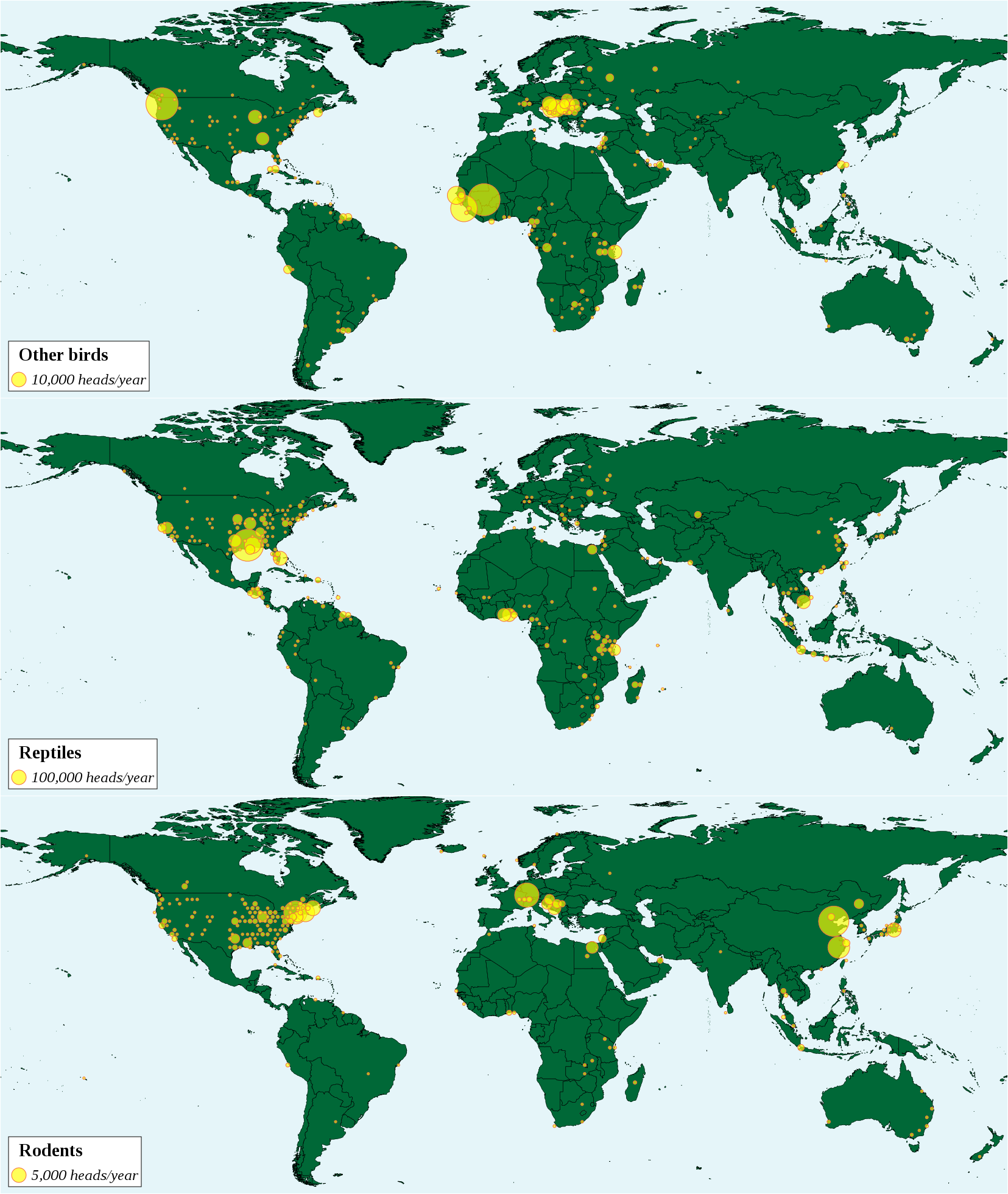

Supplement: Figure S2 — (TIF) [file pone.0070000.s004.tif]

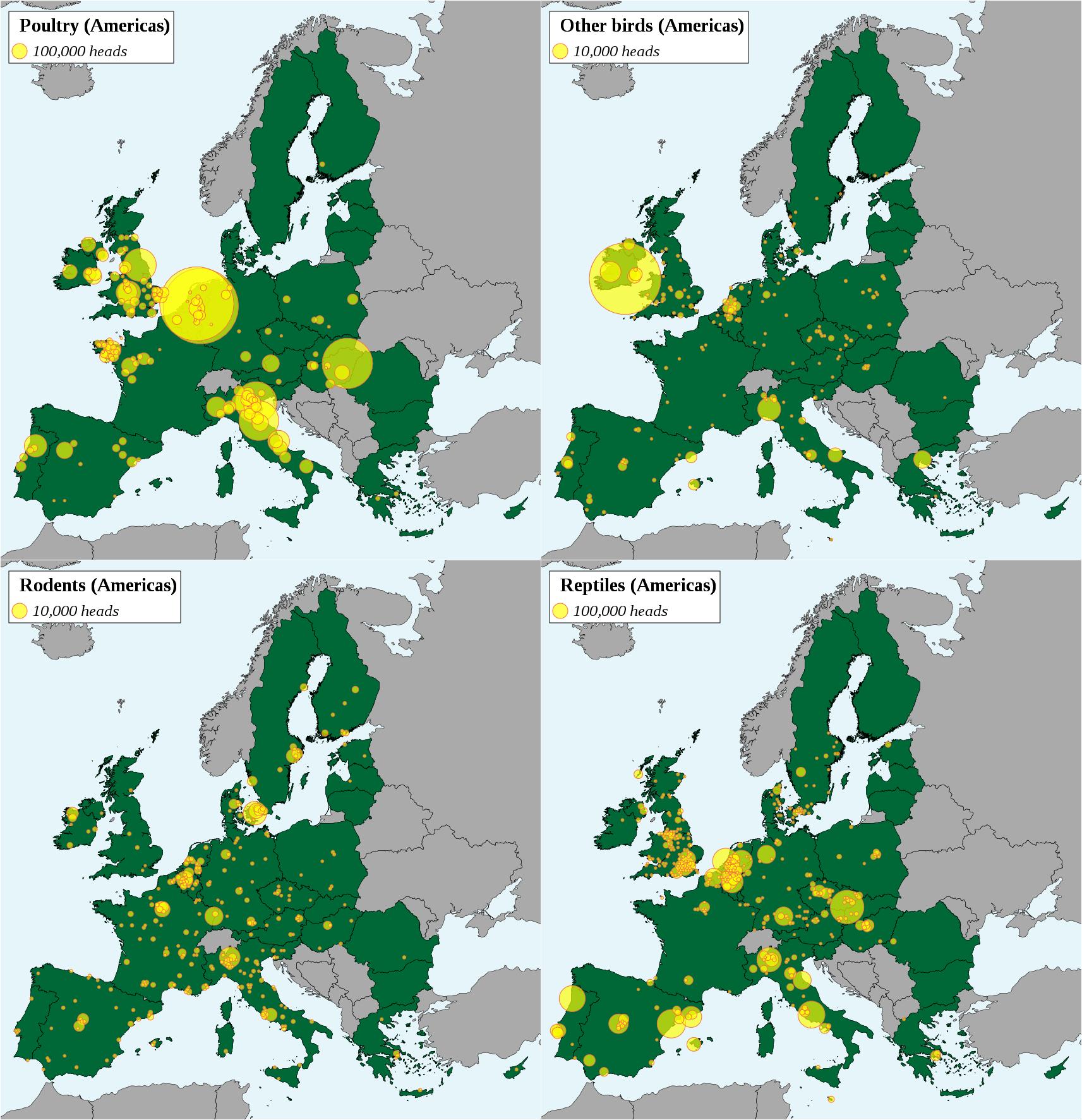

Supplement: Figure S3 — EEEV: rodents, poultry, other birds and reptiles imported from the Americas. WEEV: poultry and other birds imported from the Americas. (TIF) [file pone.0070000.s005.tif]

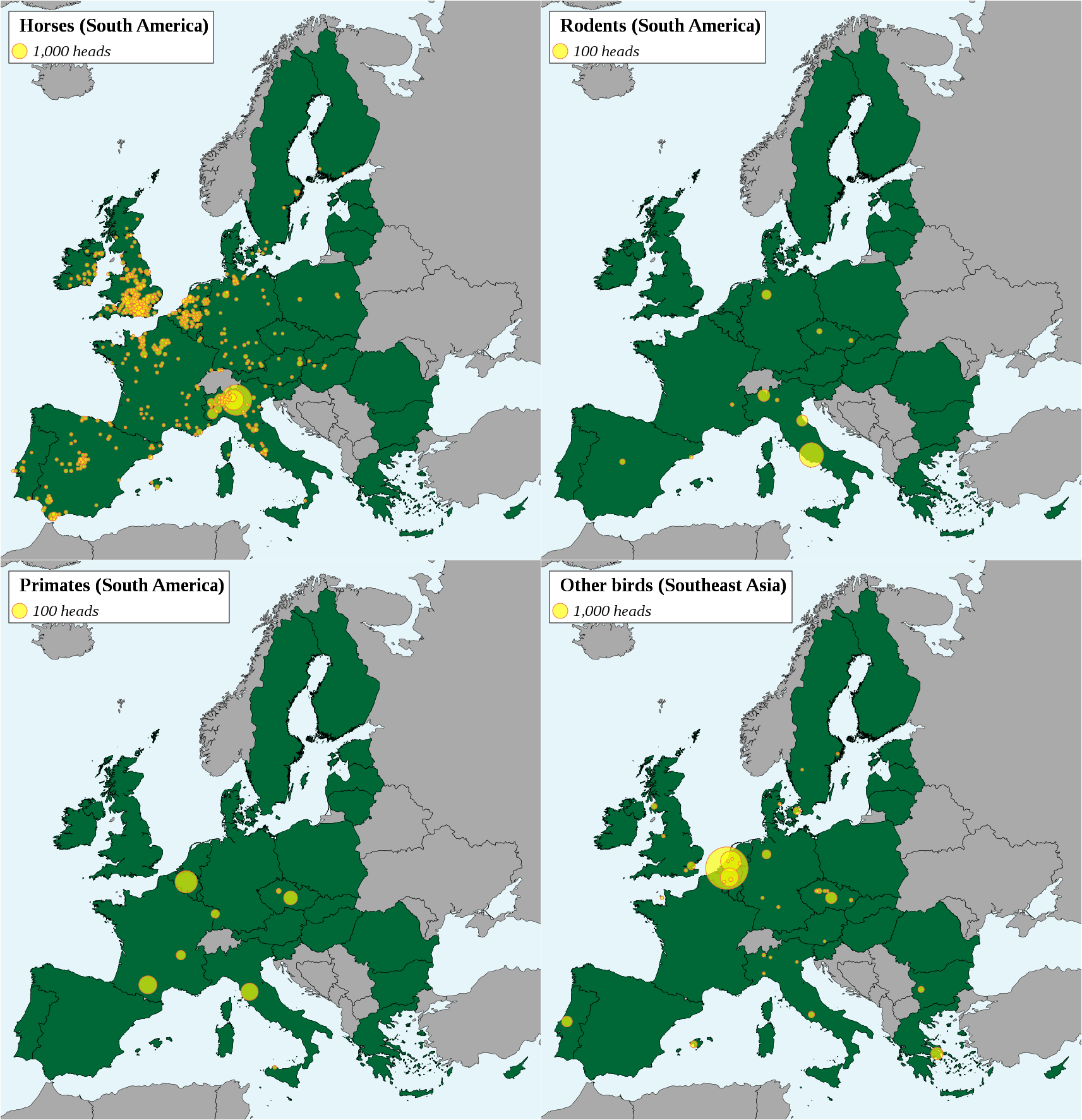

Supplement: Figure S4 — VEEV: horses, rodents, and primates imported from South America (including Central America and the Caribbean). JEV: birds other than poultry imported from Southeast Asia (including Japan, Korea, China, India and Pakistan). (TIF) [file pone.0070000.s006.tif]
